# Supplementary material for: The Identification of Cu–O–C Bond in Cu/MWCNTs Hybrid Nanocomposite by XPS and NEXAFS Spectroscopy
Source: Nanomaterials (Basel). 2021 Nov 7;11(11):2993. doi: 10.3390/nano11112993 (PMC8621448; doi:10.3390/nano11112993)
Supplement: Supplementary file 1 [file nanomaterials-11-02993-s001.zip › nanomaterials-1415654-supplementary.pdf]

nanomaterials

*Supplementary Materials*

# The Identification of Cu–O–C Bond in Cu/MWCNTs Hybrid Nanocomposite by XPS and NEXAFS Spectroscopy

**Danil V. Sivkov [1,2,*], Olga V. Petrova [2,*], Sergey V. Nekipelov [2], Alexander S. Vinogradov [1], Roman N. Skandakov [2], Sergey I. Isaenko [2], Anatoly M. Ob'edkov [3], Boris S. Kaverin [3], Ilya V. Vilkov [3], Roman I. Korolev [4] and Viktor N. Sivkov [2]**

[1] Federal State Budgetary Educational Institution of Higher Education, Saint-Petersburg State University, 199034 St. Petersburg, Russia; asvinograd@yahoo.de
[2] Komi Science Centre of the Ural Branch of the Russian Academy of Sciences, 167982 Syktyvkar, Russia; nekipelovsv@mail.ru (S.V.N.); scanick@yandex.ru (R.N.S.); isaenko@geo.komisc.ru (S.I.I.); sivkovvn@mail.ru (V.N.S.)
[3] G.A. Razuvaev Institute of Organometallic Chemistry of the Russian Academy of Sciences, 603950 Nizhny Novgorod, Russia; amo@iomc.ras.ru (A.M.O.); kaverin@iomc.ras.ru (B.S.K.); mr.vilkof@yandex.ru (I.V.V.)
[4] Pitirim Sorokin Syktyvkar State University, Syktyvkar 167001, Russia; korolev36a@gmail.com
* Correspondence: d.sivkov@spbu.ru (D.V.S.); teiou@mail.ru (O.V.P.); Tel.: +7-812-4284352 (D.V.S.); +7-821-2391461 (O.V.P.)

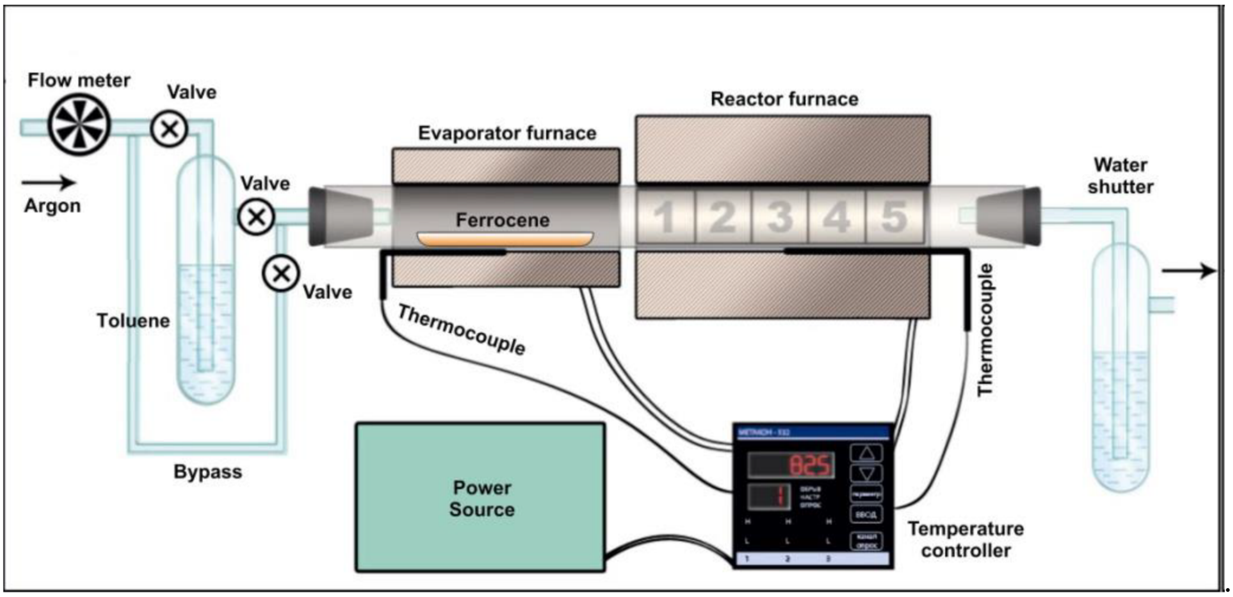


**Figure S1.** A schematic illustration of an experimental setup for growing radial-oriented aligned MWCNTs.

The schematic illustration of the experimental setup used for the synthesis of radial-oriented aligned MWCNTs is shown in Figure S1. It consists of a cylindrical quartz reactor (length — 600 mm, outer diameter — 24 mm, inner diameter — 20 mm) placed in a dual furnace system. Inside the cylindrical reactor in the pyrolysis zone 5 cylindrical quartz inserts 50 mm long with an external diameter of 19 mm and an internal diameter of 17 mm are placed. At the entrance to the reactor main chamber a system for bubbling and supplying an inert gas was implemented. To exclude the possibility of atmospheric air entering the reactor volume an outlet water shutter was used. The process was performed as follows. A quartz boat with a ferrocene was introduced into the evaporator furnace with an Ar-flow 100 sccm (standard cubic centimeters per minute) and the reactor furnace was preheated at T = 825 °C. The Evaporator furnace and the Reactor furnace with thermocouples located inside are connected to the power source through the METACON-532

temperature controller. At high temperatures (825 °C), the Reactor furnace has a uniform (± 2 °C temperature distribution only in the middle pyrolysis zone (region 3 in Figure S1), the length of which is 50 mm, and the furnace length is 250 mm. In a subsequent step, the ferrocene placed in the Evaporator furnace was warmed at a temperature of 130 °C. The argon flow 500 sccm, controlled by a high-precision gas flow meter AALBORG GFS17, entered the bubbler with toluene and transferred toluene vapor to the Evaporator furnace. Ferrocene vapors were sublimated from a quartz boat and mixed with toluene vapors in an argon stream. The vapor mixture was transferred further to the Reactor furnace. In the central zone of the Reactor furnace, the mixture of ferrocene and toluene vapors decomposed with the formation of MWCNTs radial-oriented arrays on cylindrical quartz inserts. The temperature of the Evaporator furnace, Reactor furnace, ferrocene mixture and toluene vapor, and the argon flow rate were selected so that the MWCNTs arrays obtained in the third pyrolysis zone of the Reactor furnace had equal wall thickness, along the entire length of the deposited nanotube array. In order to obtain cylindrical arrays of MWCNTs with equal walls thickness the pyrolysis temperature was 825 °C and the synthesis time – 4 h. After the synthesis, the furnaces were cooled to room temperature, the reactor was opened and the quartz insert with the MWCNTs array deposited inside was removed from the third pyrolysis zone. Then, the MWCNTs array was mechanically separated from the quartz liner and milled to a powdery homogeneous state suitable for subsequent studies and synthesis of hybrid materials based on MWCNTs.

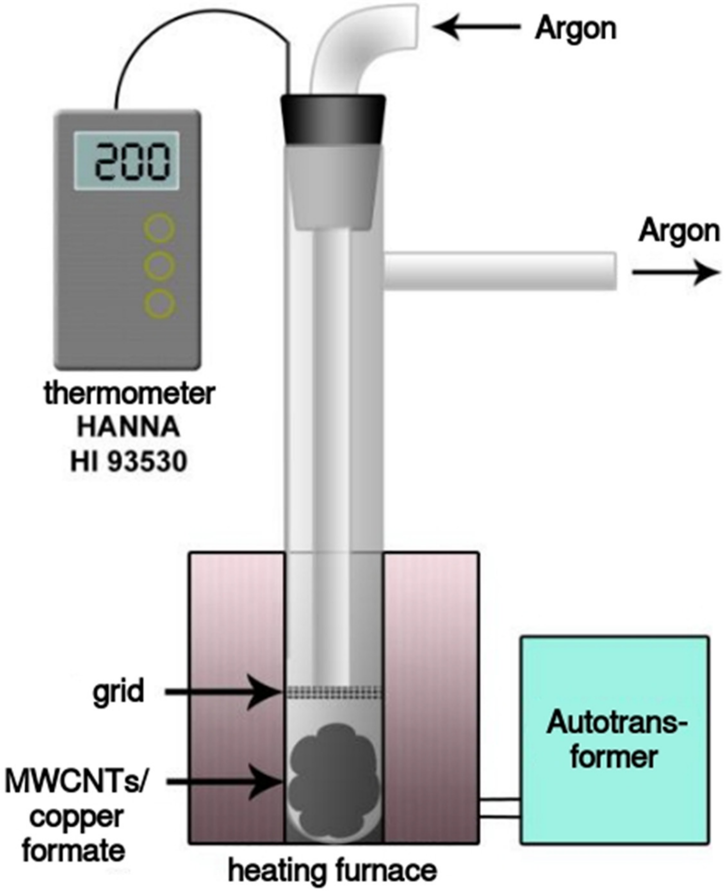


**Figure S2.** A schematic illustration of an experimental setup for the Cu/MWCNTs nanocomposite synthesis.

The schematic illustration of the experimental setup for Cu/MWCNTs nanocomposite synthesis is shown in Figure S2. The process occurs in two stages: 1) the precursor deposition on the MWCNTs surface and 2) subsequent decomposition with the formation

of copper nanoparticles. For the copper formate deposition on the surface of MWCNTs, 150 mg of copper formate was dissolved in 100 ml of distilled water by ultrasonic treatment for 1 hour. A powder of the initial MWCNTs was placed in the resulting solution. To obtain nanocomposites, its weight ratio to copper formate was chosen equal to 1: 0.5, 1: 1, 1: 5, and 1:10. The MWCNTs dispersion in the copper formate aqueous solution occurred during treatment in an ultrasonic bath for three hours. Then the resulting mixture of MWCNTs and copper formate in water was placed in a SNOL-58/350 drying oven at a temperature of 95 °C until the liquid was completely removed. The resulting dry powdery precipitate of MWCNTs with copper formate deposited on their surface was used to synthesize a hybrid material.

The catalyst was synthesized in a Pyrex glass reactor at 473 °K in a flow of high-purity argon. The MWCNTs with copper formate deposited on their surface were placed on the bottom of the quartz reactor and the temperature in the pyrolysis zone was gradually raised to 473 °K in an argon flow. Copper was deposited onto the MWCNT surface during the copper formate pyrolysis to obtain a Cu-MWCNT hybrid material according to the scheme:

$$(H\text{–}COO)_2Cu \rightarrow Cu + 2CO_2 + H_2$$

Pyrolysis products of copper formate in an argon flow were removed from the reactor through a water seal. After the copper formate pyrolysis, the reactor was cooled and the resulting catalyst was removed and was stored in a volume filled with high-purity argon.

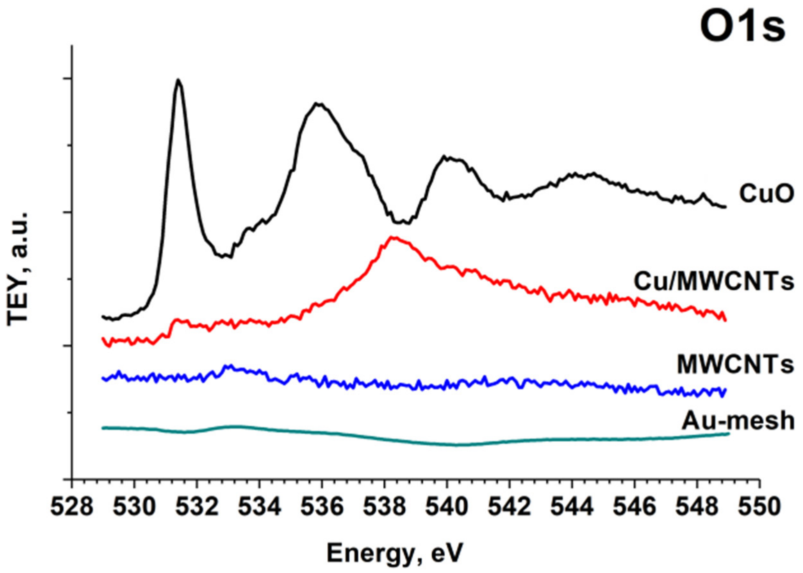


**Figure S3.** The TEY signals in the O 1s NEXAFS region of the initial MWCNTs, Cu/MWCNTs nanocomposite, CuO oxide and Au-mesh.

When studying the O1s absorption spectra fine structure in the atomic ionization threshold, it is necessary to take into account the presence of an additional structure in the incident radiation spectrum due to oxide layer and oxygen-containing compounds on the reflecting metal coatings of the monochromator optical elements. The studied spectra normalization to the incident radiation intensity (typically measured by recording the TEY signal of a gold grid or plate) can lead to strong distortions of the O 1s NEXAFS spectra. Figure S3 clearly shows that the structure in the incident synchrotron radiation beam at the NanoPES station output (the Kurchatov synchrotron radiation source) is insignificant and does not distort the O 1s NEXAFS spectra of the compounds under study.

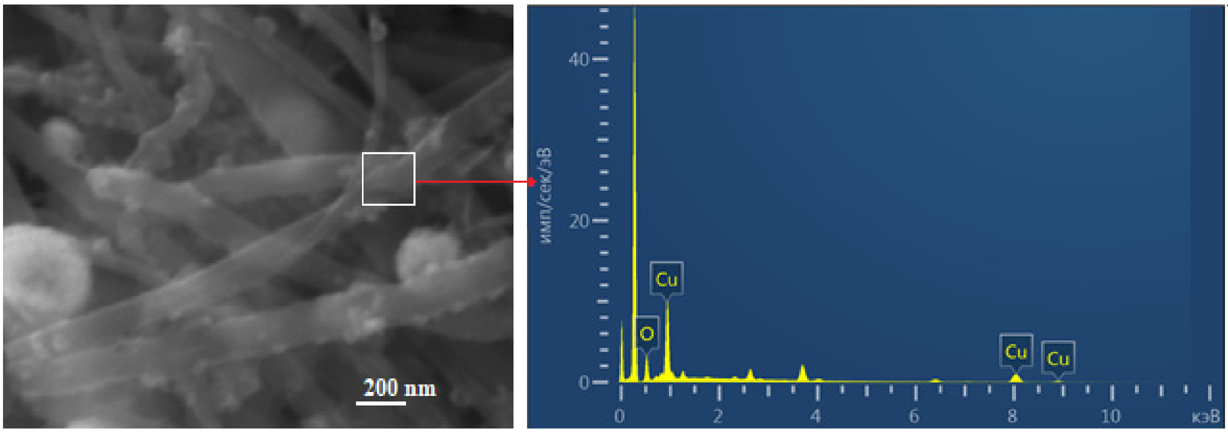


**Figure S4.** Electronic microscopy image of the Cu/MWCNTs nanocomposite and EDS review of the *uncoated nanotube parts.*

In Figure S4 the SEM image of nanocomposite region free of Cu-coating and its EDS spectrum are shown. EDS data shows the peaks of carbon, oxygen and copper with 87.2%: 9.0%: 3.8% atomic ratios, respectively. Taking into account that copper in the nanocomposite is mainly in the form of CuO oxide, the content of oxygen atoms in the form of carbon oxides can be estimated as ~5%, and is approximately 2 times higher than in the initial nanotube.

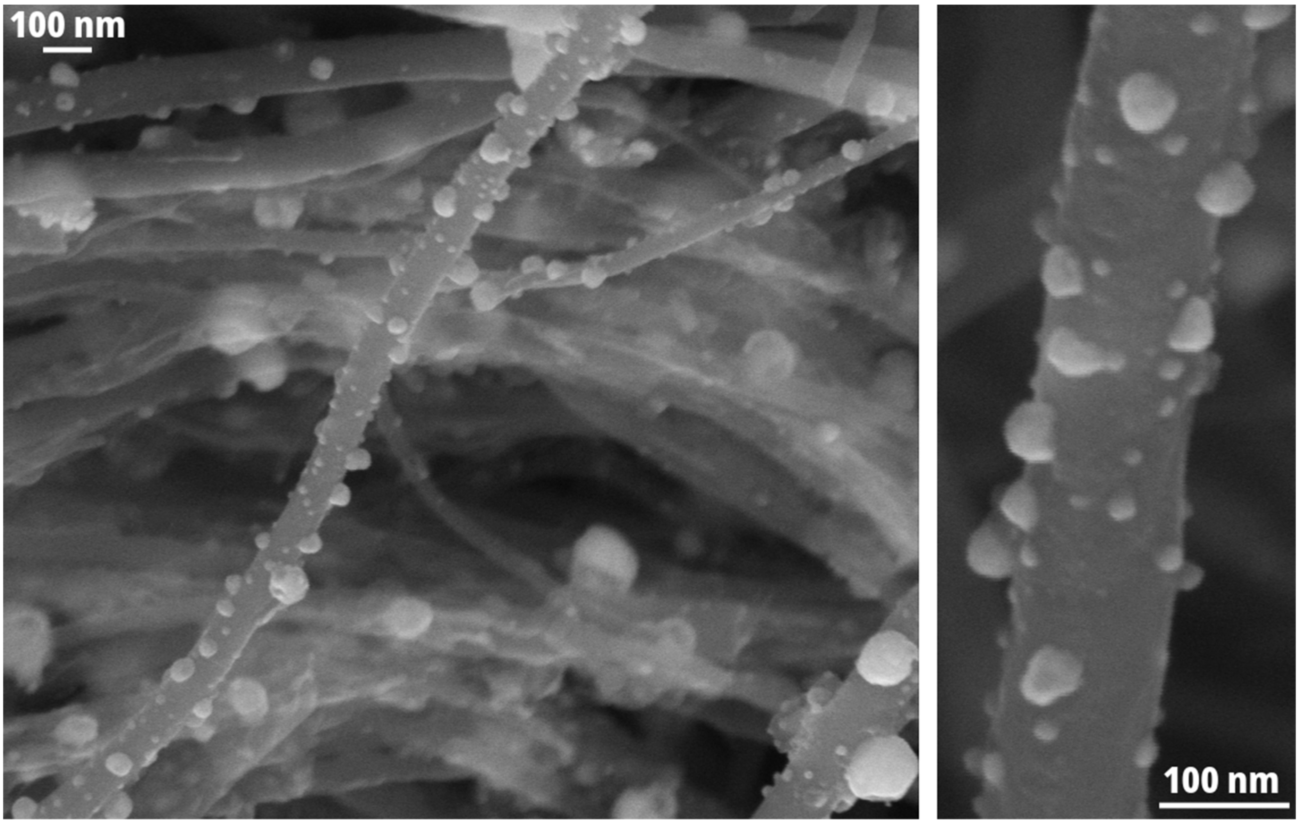


**Figure S5.** SEM images of the Cu/MWCNTs nanocomposite.

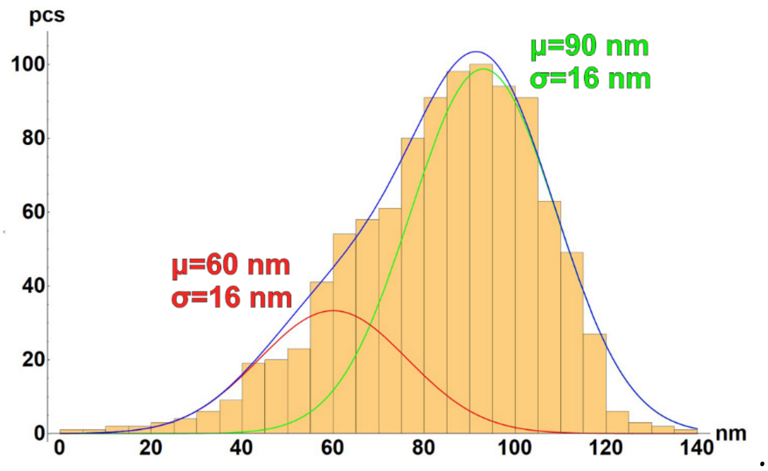


**Figure S6.** The bimodal distribution of the MWCNT diameters.

The MWCNT diameters have a bimodal distribution, which is well described by normal distributions with the following parameters: 1) μ = 60 nm, σ = 16 nm, 2) μ = 90 nm, σ = 16 nm (μ is the median, σ is the variance (half-width at half-height)) (Figure S6).

The MWCNTs samples obtained as a result of synthesis are oriented arrays of nanotubes. The order of length of most MWCNTs is the same as the thickness of the resulting arrays ~1 mm. However, both for practical application and in the technology of obtaining hybrid nanomaterials, it is necessary to reduce the average length of MWCNTs to acceptable one. After grinding, the length of MWCNTs is a random variable and obeys the normal distribution, but in the approximation, it is also well described by a continuous uniform distribution.

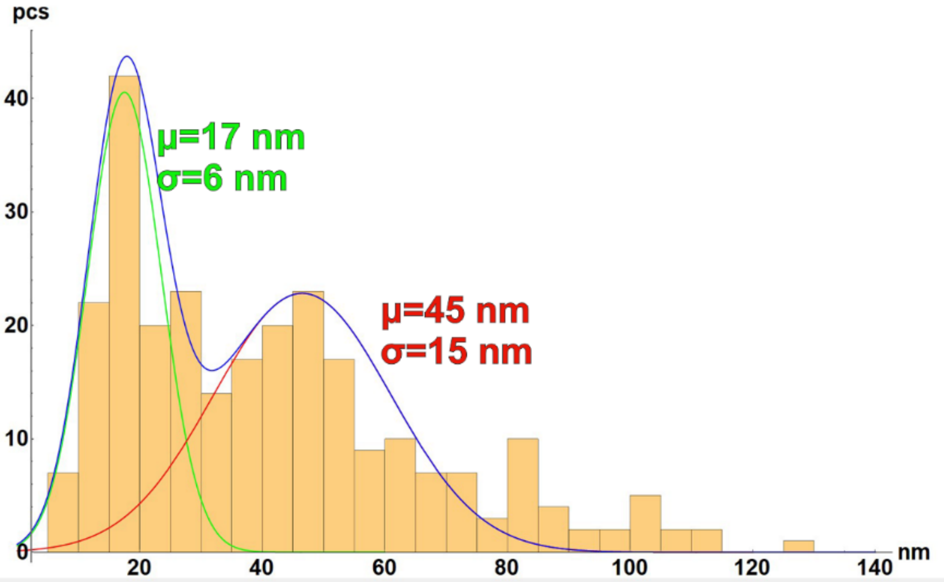


**Figure S7.** The bimodal distribution of the Cu containing nanoparticle diameters.

The distribution of Cu containing nanoparticles by diameter is also bimodal, and well described by normal distributions with the following parameters: 1) μ = 17 nm, σ = 6 nm, 2) μ = 45 nm, σ = 15 nm (μ is the median, σ is the variance (half-width at half-height)) (Figure S7).

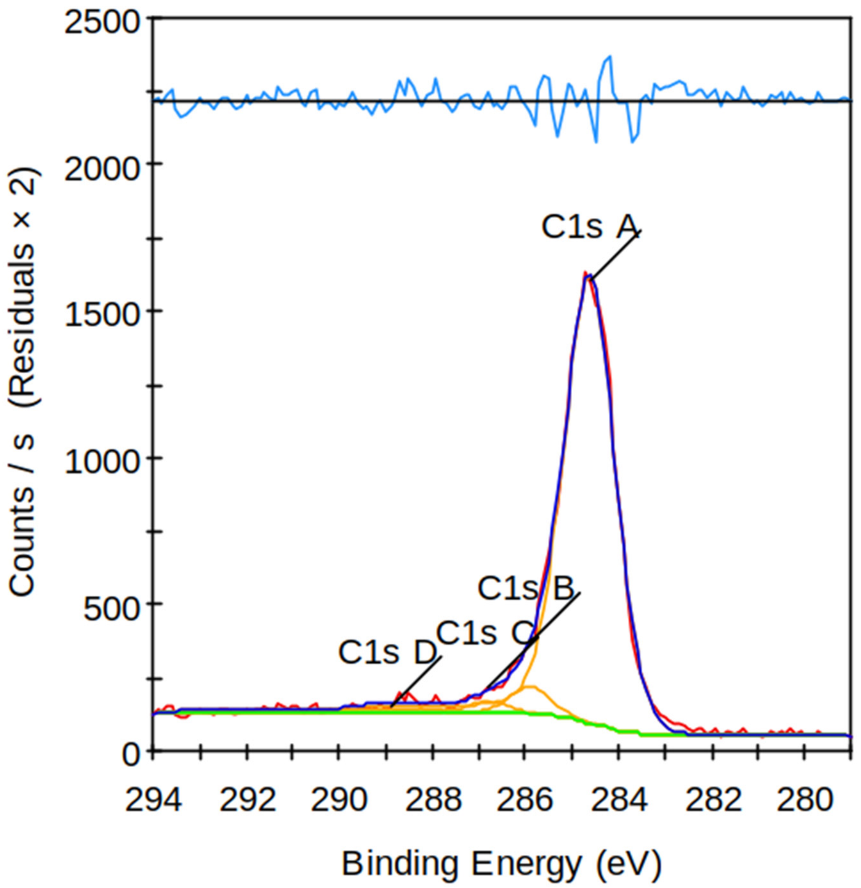


**Figure S8.** Spectral decomposition (fitting) of C 1s XPS spectra of Cu/MWCNTs nanocomposite.

The Cu/MWCNTs C 1s XPS spectra decomposition (Figure S8) was performed using the ESCALAB 250 Xi spectrometer software (Avantage V5.9904, Thermo Fisher Scientific, Waltham, MA, USA). Table S1 contain the corresponding parameters of decomposition.

**Table S1.** The paremeters of the spectral decomposition of C 1s XPS spectra of Cu/MWCNTs nanocomposite.

| Name | Peak BE | FWHM (eV) | Area (CPS*eV) | L/G mix (%) | Tail mix (%) | Tail Height (%) | Tail Exponent |
|---|---|---|---|---|---|---|---|
| **C1s A** | 284,59 | 1,29 | 2219,96 | 30 | 94.14 | 0 | 0.054 |
| **C1s D** | 288,90 | 1,52 | 33,33 | 30 | 100 | 0 | 0 |
| **C1s B** | 285,90 | 0,99 | 99,68 | 30 | 100 | 0 | 0 |
| **C1s C** | 286,81 | 0,99 | 37,58 | 30 | 100 | 0 | 0 |
